# Exploring Educational Planning, Teacher Beliefs, and Teacher Practices During the Pandemic: A Study of Science and Technology-Based Universities in China

**DOI:** 10.3389/fpsyg.2022.903244

**Published:** 2022-04-29

**Authors:** Yang Gao, Gang Zeng, Yongliang Wang, Amir Aziz Khan, Xiaochen Wang

**Affiliations:** ^1^School of Foreign Languages, Dalian Maritime University, Dalian, China; ^2^School of College English Teaching and Research, Henan University, Kaifeng, China

**Keywords:** science and technology universities, educational policies, teacher beliefs and practices, a mixed-methods study, COVID-19

## Abstract

With the spread of the COVID-19 pandemic worldwide, university teachers are coping with and adjusting to online teaching platforms. In this concurrent mixed-methods study, 10 science and technology universities as the research sites were first chosen, and educational planning in these sites during the pandemic was examined; then, eight selected teacher participants in these sites were interviewed to report how their beliefs and practices changed during the pandemic echoing the examined educational planning. The results show that educational planning and policies assisted teachers in accommodating the new demands and changes during the pandemic; teachers' beliefs and practices generally echoed the educational planning and policies, with certain tensions still existing. The discussion part of the study is centered around emergency remote teaching and planning, tensions between teacher beliefs and practices, and the shift from emergency remote teaching to regular, sustainable online schooling. The study provides administrators and teacher educators with insights on how emergency remote teaching can be planned and implemented during an unprecedented time.

## Introduction

The COVID-19 pandemic has been widely spreading across the globe since the beginning of 2020. According to *Worldmeter*, the global statistics include 309,914,150 cases by 6:30 am, January 11, 2022; within a single day, the number of confirmed cases has reached as high as 2,228,176.

The unexpected pandemic has caused massive damage, suspension, and even death to all walks of life; education has been severely affected. Most, if not all, schools have had to move their schooling online in response to the pandemic. In some countries, governments, together with corresponding Ministries of Education (MOE), issued timely policies and support to schools, institutes, and universities to facilitate their academic resilience; faculty members, professors, and instructors then actively took the initiative and responded to these policies with full support. According to a comparative study between China and the United States (Wu and Guo, 2021), which surveyed almost 1,000 institutes and universities in these two countries (451 in China vs. 308 in the United States), most faculty members in higher education confirmed the positive effects of these policies and appreciated the support from the related parties, typically through the first wave of the pandemic (around the beginning of 2020 to July 2020).

While studies overall confirmed the educational planning policies from different institutes and universities, they failed to provide specific details on how teachers have perceived these policies and acted accordingly. Given that the global pandemic is still ongoing, we must prepare for a long-term struggle against the virus. Therefore, exploring teachers' beliefs and practices regarding their educational policies and planning is a necessity for the sake of bettering our online teaching and catering to our new unpredictable reality.

In the current mixed-method study, we first traced the educational planning policies of 10 different universities in China and explored how teachers had perceived and acted toward these policies during the first wave of the pandemic (January 2020 to July 2020), when most universities had ended their semesters. In other words, we explored how national and institutional policies during an unprecedented time had informed pedagogical decisions about designing and implementing online courses and examined whether teachers or instructors indeed took these decisions and how they had perceived their results. Therefore, we selected science and technology universities as our target universities and aimed to explore answers to the following two research questions:

- How have the educational planning and policies informed the design and implementation of instruction during the pandemic?
- What are teachers' beliefs and practices regarding these educational planning policies?

## Literature Review

### From E-Learning to Emergency Remote Teaching

E-learning, or online learning, has caused a shift in the behaviors and functions of both teachers and students and also a change in the schooling environment. We roughly define all web-based education, digital learning, interactive learning, computer-assisted teaching, and internet-based learning as part of or one type of e-learning (Lara et al., 2020). Studies confirm the benefits of e-learning, which includes, but is not limited to, real-time communication and interaction between teachers and students and advanced development of educational or instructional tools (e.g., Samir et al., 2014). However, studies also report the disadvantages of e-learning, arguing it requires teachers and students to learn additional technological or operational knowledge together with the content area knowledge (e.g., Hodges et al., 2020). While the argument over e-learning remains in a quandary, it is widely acknowledged during disaster times that e-learning facilitates instruction during unpredictable times.

E-learning is thus one of the products or occurrences that help people deal with education interruption. However, differences still exist between typical e-learning and emergency remote teaching (ERT). The primary difference between e-learning and ERT is about planning. Hodges et al. (2020) stated that e-learning is planned from the beginning and deliberately designed to be implemented online, whereas ERT is a temporary shift of instructional delivery to an alternate delivery mode due to crisis circumstances. Ever since SARS and H1N1, ERT has been confirmed as a powerful alternative to face-to-face instruction. For example, Meyer and Wilson (2011) explore how ERT was included in an institution's plans to handle instructional emergencies during H1N1. Findings show that two-thirds of them did not include any reference to online learning as a way to continue coursework while one-third included suggestions to faculty to find alternative ways of delivering courses, using technology or specific tools to do so; however, only one of these institutions actually mentioned online learning. In another study on post-earthquake emergencies, Mackey et al. (2012) report how ERT helped teachers cope with instructional challenges or demands in the aftermath of an earthquake. Findings show that organizational understanding and capability for blended and online learning already exists, along with appropriate infrastructure as well as technical and student support mechanisms to facilitate its use. Cahyadi et al. (2022) also reported their findings on the ERT evaluation during COVID-19 in Indonesia. In addition to the description of procedures and various factors informing the ERT, they also advocated that educational planning during the pandemic should go through the *simplicity, flexibility*, and *empathy* principles. In another study, Trust and Whalen (2020) advocated infusing the entire curriculum with high quality and quantity technology experiences and providing teachers with unstructured professional development through mentoring and online forums. The findings of these studies paved the way for teacher educators on how to better prepare and support teachers for teaching remotely in times of need.

### Educational Planning and Policies During the Pandemic

Educational planning can be defined as the planning process that helps prioritize goals in a certain educational system and then adequately realizes these goals with the available human or material resources (Norogbo, 2004). As stated above, ERT is often spontaneous without expectation and thus in need of great planning during emergency or disaster times. With the spread of COVID-19, educational planning thus becomes overwhelmingly important in guiding faculty and instructors to design curricula, deliver instructions, manage classrooms, and maintain routine schooling. Therefore, studying educational planning at such a crucial time becomes necessary. Quite a few studies have already informed readers of the educational planning process during the pandemic, both globally and regionally.

Globally, the Organization for Economic Co-operation and Development (OECD) has released a number of reports on policy responses during the pandemic. Specifically, it issued its first advocated module in late March 2020, i.e., “A framework to guide an education response to the COVID-19 Pandemic as a tool to support education leaders smoothing over challenges and difficulties in maintaining a regular schooling during the pandemic.” The framework was based on a cross-national survey assessing educational needs, priorities, implementation challenges, emerging responses and strategies to help monitor the emerging needs and evolutional responses from different stakeholders, educators, and in-service teachers in the education sector. OECD also released another module, providing the reader, typically teachers, with possible online educational resources to facilitate the continuity of teaching and learning during the pandemic around the world. Different modules as guides for educational planning have been sequentially organized and drafted at the time of this writing.

Regionally, different countries also contribute their own studies to the existing literature in guiding educators and teachers to maintain routine schooling during this unprecedented time. For example, with the outbreak of COVID-19, the Chinese government launched an emergency policy called “Suspending Classes Without Stopping Learning” to fight against the spread of the epidemic by suspending offline teaching at schools and turning to online education (Zhang et al., 2020). On this very note, the government carried out the following measures to implement the policy: integrating national resources and planning at the top-level, training teachers, enabling local authorities and schools to carry out online teaching in line with local conditions, formulating guidelines to prepare for smooth transition back to normal offline education after the epidemic and working out a plan for reopening schools after the pandemic.

Likewise, Masri and Sabzalieva (2020) investigate educational planning from the federal and local governments and policy responses from higher education institutes in Canada during COVID-19. Their study found that support for colleges and universities was dispersed and uncoordinated in the very first 2 months; however, plans to support research initiatives and students were gradually announced and delivered. There was some alignment between educational planning and government support, although the Ontario provincial government did not play a vital role in shaping an effective higher education response during the first wave of COVID-19.

In Nigeria, the Federal Ministry of Education (FME) issued its COVID-19 contingency plan and its operational guidance note as part of its educational planning during the pandemic. The contingency plan includes the overall objective for intervention, the specific objectives related to education, and the protection of mainstream principles for education. Ogunode et al. (2021) point out that COVID-19 affected education planning in Nigeria and led to suspensions of planned programs, school and lesson planning, and data collection of education programs. To ensure even in the midst of the pandemic that education still continues, all educational planners in all ministries and agencies of education were to be trained on how to use information and communication technologies (ICT) facilities to carry out planning functions while ICT facilities were to be provided for all educational planners in the country.

Ismail et al. (2021) investigated how educational institutions initiated educational program planning to ensure the quality of education during the pandemic in Indonesia. They argue that educational planning should work for educational policies that support the fostering and development of learners' intelligence. They also advocate through their content analysis study that teacher professionalism and competence, optimizing the quality of educational institutions and maximally implementing National Education Standards should be seriously considered when setting educational policies.

### Teachers' Beliefs and Practices During COVID-19

*Teacher beliefs* is a key concept in studying teacher practices and professional development, typically in the disaster times. Gao (2021a) stated teacher beliefs are in nature complex, non-linear, and unpredictable. The belief system may include different theoretical orientations of teachers' subject matter, matricing in different forms and informing teachers of their practices.

The concept of teacher beliefs and perceptions has been widely explored during the COVID-19 pandemic, typically those beliefs and perceptions over educational planning, instructional approaches, classroom modes and final exams. For example, Merga et al. (2021) explore teacher perception of the COVID-19 impact on writing instruction in Australia and report negative impacts in context and home affordances, typically detailing the uneven distribution of parental and technological resources at home. However, some participants in their study reported an unexpected positive impact on collaboration between educators and education systems.

Another change in teacher beliefs and perceptions during the pandemic is highlighted in Yadav et al. (2021), which focuses on teacher perceptions of flipped classrooms during the COVID-19 pandemic and reports that faculty still preferred face-to-face instruction to a flipped classroom, due to a lack of student participation and feedback in a flipped classroom. Other challenges, including internet access and technical difficulties, were also reported to affect faculty members in offering high-quality teaching during the pandemic.

In addition, Campbell and Harris (2021) focus on teacher perception of the educational planning policies and a principal's support during COVID-19. Support from principals was acknowledged and confirmed by the surveyed sample, as principals provided timely training to teachers and supervised professional development. Campbell and Harris' findings are consistent with other studies (e.g., Gao et al., 2021).

While existing literature attempts to study educational planning and teacher perceptions over e-learning during COVID-19, there is still a dearth of literature on tensions between educational planning policies and teacher perceptions over these policies, especially ones using a mixed-methods approach. Most of the existing literature adopts quantitative methods, with surveys being the primary tool in data collection. However, the quantitative nature of this tool fails to provide specific exploration or detailed descriptions of teacher perception. Therefore, the current study aims at addressing the gap by adopting a mixed-method approach and typically attempts to explore teacher perception at the qualitative stage.

## Research Methodology

### Research Design: A Concurrent Mixed-Methods Study

In this present study, we adopt a concurrent mixed-method design (Creswell, 2014). When choosing our research method, we considered whether the design fits our research aim, purposes, and questions. The primary purpose of this study is 2-fold: we first aim to analyze the educational planning policies among the selected universities and to examine how these planning policies might facilitate instruction during the pandemic; we then aim to explore teacher perceptions of these educational planning policies to see if any tensions exist between teacher perception and actual practice. The following chart describes how the design was structured for the entire study.

We mapped out two stages for this study according to our design. For the first stage, we used policy analysis as the methodology to study how general policies inform alternatives and consequences over a given time (Walker and Europe, 2000). Policy analysis equips researchers with a way for understanding how and why governments or policymakers enact certain policies and how the effects from these policies would be (Browne et al., 2019). Conducting a policy analysis ensures researchers go through a systematic process to choose the policy option that may be best for a certain situation, the tough one in particular. Generally, the policy analysis method may fall into a trifold classification including *traditional, mainstream* and *interpretive* policy analyses, due to different epistemologies and ontologies behind the three analyses (Colebatch, 2006; Bacchi, 2009). A traditional policy analysis involves deploying a rational comprehensive approach to problem solving, in a world that is objectively knowable. A traditional policy analysis falls into a positivist paradigm that regards the world as objectively knowable, and the issuing policy should be in a rational, linear manner. It thus employs rational, comprehensive, but static approaches or policies to tackle problems. A mainstream policy analysis is interactive and systemic, going through typically agenda-setting, policy processes, policy networks, and governance process. On this very note, policy is conceptualized as “the interaction of values, interests and resources guided through institutions and mediated through politics” (Davis et al., 1993, p. 15). An interpretive policy analysis derives from the interpretive turn during the late nineteenth century and complements the other two approaches. It focuses not only on meanings but also on how researchers approach, generate and explain these meanings (Yanow, 2007; Gao, 2020). However, the complex, pragmatist world has enabled scholars to use a mixture of the analyses to tackle the real problems, thus making the boundaries among the three analyses sometimes blurred (Gao, 2020). In this current study, we adopted the mixed policy analyses method which combines mainstream policy analysis and interpretive policy analysis.

With such a methodological framework, we traced the issuing of official policies and documents from both national and institutional levels and analyzed these policies and documents from both macro and micro perspectives. Our macro analysis delves into connections and tensions between national and institutional policies and how policies were delivered in a hierarchical and timely order. Our micro analysis then explores how these policies and subsequent policies emerging from these policies helped to maintain the operation of online courses during the pandemic. The second stage involved exploring teacher perception and practices about these educational planning policies. To make our study manageable, we include only eight purposive participants from each of our selected sites. We used a semi-structured survey to interview participants and then analyzed their interview transcripts.

### Sample Sites and Participants

We selected 10 universities for the study according to different geographic locations (regions), types of the universities (i.e., research or teaching universities), and also national rankings (i.e., Shanghai Ranking Consultancy). While we attempted to employ random sampling, in the end we went through convivence sampling due to time constraints and study manageability. Therefore, we selected five research-based universities and five teaching-based universities. Like the university dichotomy in other countries, a research-based university in China is primarily focused on research development and sectioned into subdivisions within a particular subject. Most of these universities are rich in research grants and strive for boosting their reputation and ranks by their research achievement. In contrast, teaching universities are more on the side to develop quality teaching, with more undergraduate students than graduate students. All universities in the study were ranked from 1 to 10, indicating a gradual decrease in national rankings; site 1 was ranked approximately within the top 20 in the nation and the last one listed was not included in the ranking system. Four of the selected universities were from northeast China, two from central eastern China, two from central China, and the final two from northern China. The basic information of the selected sites is listed below in Table 1.

**Table 1:** Information of the selected sites.

	**1**	**2**	**3**	**4**	**5**	**6**	**7**	**8**	**9**	**10**
Region	N	NE	NE	CE	NE	CE	NE	N	C	C
Type	R	R	R	R	R	T	T	T	T	T

*N, north; NE, northeast; CE, central east; C, central*.

In the second stage, we further interviewed eight participants from the selected universities. The participants were purposefully selected according to certain criteria, including but not limited to teacher willingness to participate, teacher availability for classroom observation, and the manageability of the study. Specifically, we included five female participants and three male participants. All had been working in their respective sites for two to 14 years at the time the study was conducted. Their ages ranged from 30 to 40. Table 2 lists the biographical information of the six purposive participants.

**Table 2:** Biographical information of the participants.

**Participant**	Gender*	**Age**	**Years of teaching experience**	**Subject or major taught**
1	F	32	6	College English/non-English major
2	F	39	14	College English/non-English major
3	F	39	14	Intensive English/English major
4	M	38	14	Writing/English major
5	M	36	10	Advanced English/English major
6	F	30	2	Writing/English major
7	F	40	10	College English/non-English major
8	M	33	6	College English/non-English major

* *M, male; F, female*.

### Data Collection and Analysis

We collected and analyzed data according to the two stages scheduled in the study. In the first stage, we collected official documents, policies, and news from different sources. Specifically, we divided these sources into top level, mid-level, and bottom level sources: the top level included information from the central government, the MOE, and/or provincial/municipal governments; the mid- level included information from institutional policies, news, and notices; the bottom level included information from schools, departments, and/or teacher teams. The following table summarizes all these documents (see Table 3).

**Table 3:** Official policies from different sources.

U*	Central Gov. and MOE*	Prov/Muni Gov.s*	EHB*	AAO*	HRO*	IT&SO*	TQAT*	Schl/Dept*
1	12	7	22	1	5	3	1	1
2	14	5	24	2	4	2	1	2
3	17	2	19	13	16	8	8	2
4	12	4	11	8	11	3	2	1
5	8	3	7	1	4	4	1	2
6	9	4	30	11	6	3	1	3
7	14	7	41	4	4	2	2	2
8	15	14	11	2	8	2	2	3
9	11	6	10	3	4	3	1	1
10	12	6	12	2	5	1	1	1

* U, university; Central Gov. and MOE, central government and the Ministry of Education; Prov/Muni Gov.s, provincial and municipal governments; EHB, emergency handing board; AAO, academic affairs office; HRO, human resources office; IT&SO, information technology and security office; TQAT, teaching quality assurance team; Schl/Dept^*^, school/department.

In the second stage, we interviewed the eight participants, transcribed the interviews, and then analyzed the transcripts. Using a grounded theory method, we started with open coding followed by axial coding. In several cases, we went back to our interviewees or participants to further solicit answers to a handful of questions. By doing so, we made our coding systemic, and our descriptions saturated. Specifically, we collected all the interview notes loosely and transcribed them in excel forms. We then highlighted these transcribed excerpts with different colors, indicating different thematic categories. We labeled these categories and then grouped the excerpts from the interviews back and forth. These labeled categories then paved the way for our axial coding, which served for generating related theories (Creswell, 2012).

It is worth mentioning that ethical considerations were seriously taken into consideration when conducting this empirical study. Specifically, we delivered copies of consent forms to participants and informed them of the research objectives and confidentiality of their answers. With the participants' signed consent forms, we then guided the participants through the whole process.

## Findings

**Figure 1:**
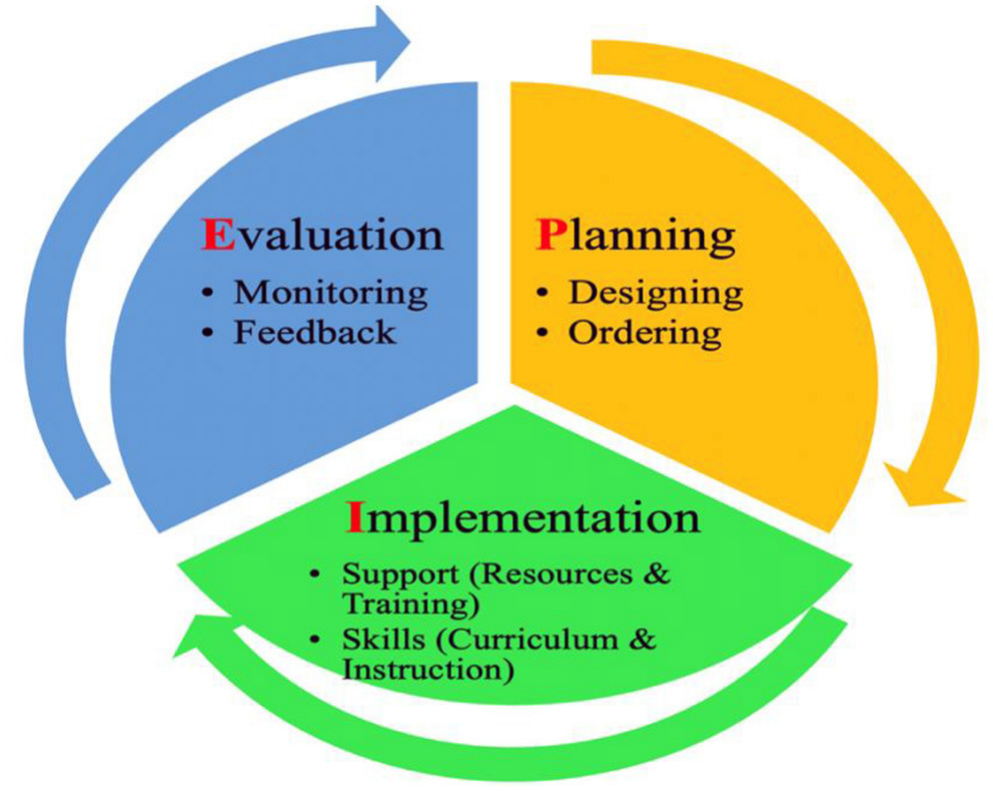
PIE education planning framework.

We found the educational planning policies had informed the design and implementation of instruction among the sampled universities during the pandemic, typically during the first round of COVID-19, prior to the Delta and Omicron outbreaks. Decision makers or stakeholders went through a planning, implementation, and evaluation (PIE) process. Specifically, in the planning stage, political and administrative parties in the country, including the MOE, were responsible for drafting and issuing the overarching or dominant policies guiding universities and institutes across the country (including the sample sites in this study); during the implementation stage, universities or institutes either organized an emergency handling board or appointed some departments or affiliates to plan for specific, detailed initiatives during the emergency; during the evaluation stage, certain departments were in charge of evaluating emergency instructions to ensure teaching quality. The stages nested in the process are listed in Figure 1.

Metaphorically, we compared the process of educational planning and policies (PIE) to the process of making and tasting a pie: the planning stage is more like how we plan and decide on the pie; the implementation stage resembles how we ask for protein (resources) and follow the recipe (training); the evaluation stage is how we taste (monitor) and comment on (give feedback to) the pie. We provide a detailed explanation on the process below.

### Baking the PIE: Educational Planning and Policies

#### The Planning Stage: How Policy Makers Design and Issue the Policies

*Overall Policies Designed and Delivered in a Hierarchal Process*. Gao et al. (2021) report:

“In a typical collectivist culture, political and institutional policies mandated teachers to uniform their designs and practices, and… policies (thus) went through a hierarchal process from the governments, either national or local, to the institutes, and then to the individual school or departments” (p. 445).

In the study, all kinds of policies were designed and delivered in a hierarchical process, from the central government and the MOE to local governments (either provincial or municipal) and finally, to individual universities or institutes. Specifically, it was the central government that had set up the overall tone and announced national decisions on how to handle the emergency and deliver lessons during the pandemic. The MOE then issued the ultimate guiding principle of “campus closed; classes continue (*CCCC*)” to all levels of schooling, including higher education institutions (Gao et al., 2021). The principle was then forwarded to provincial and municipal governments, and finally to the bureau or department in charge of educational affairs.

Then, the official policy was taken from the local governments, bureaus, and city halls to individual universities and institutes. Some local governments made additions to central government policies or provided clarifications or amendments to the *CCCC*. Furthermore, these local governments provided individual institutes additional wiggle room to interpret the policy without additional explanation. In the study, we found that all universities followed this track and followed orders and policies given by the central government and the MOE. However, some universities, e.g., sites 1, 2, and 5, forwarded the policies immediately and provided their faculty members and instructors with additional information or explanation later. Other universities, e.g., sites 3 and 8, simply forwarded central government policies without giving their instructors' further information or analyses of the policies. Even within the same province, sites 2 and 6 responded to and analyzed the policies differently. Site 6 closely followed the provincial government policies and distributed all timely news and policies pertinent to the pandemic; site 2, however, selected and forwarded only major, important policies.

Next, the Presidential Board or the General Affairs Office at most individual universities or institutes organized a meeting to set up an Emergency Handling Board (EHB) specifically designed or organized to handle all issues or plans for schooling during the pandemic. Generally, EHBs consisted of presidents, deans from related schools or departments, and deans or directors from major administrative departments (including but not limited to Academic Affairs Office and Human Resources Office). In this study, all universities or institutes set up their EHBs around late January, between January 19 and January 27, 2020. For example, site 4 set up its EHB on January 27, 2020, and site 5 on January 19, 2020. Overall, all universities responded to the emergency and set up their own EHBs in a timely manner, 3 weeks or even 1 month prior to the coming spring semester. The delivery of policies and execution of timely emergency action guaranteed the smooth operation of online instruction and regular schooling.

#### Specific Collaborative Policies to Facilitate Online Instruction

In this study, we found that different administrative offices within their universities collaborated and planned for online instruction during the pandemic, following primary direction from the EHB. Taking site 5 as one example, with guidance from the EHB, different administrative departments or offices worked together to smooth over difficulties and challenges to ensure the integrity of instructional practices.

**Figure 2:**
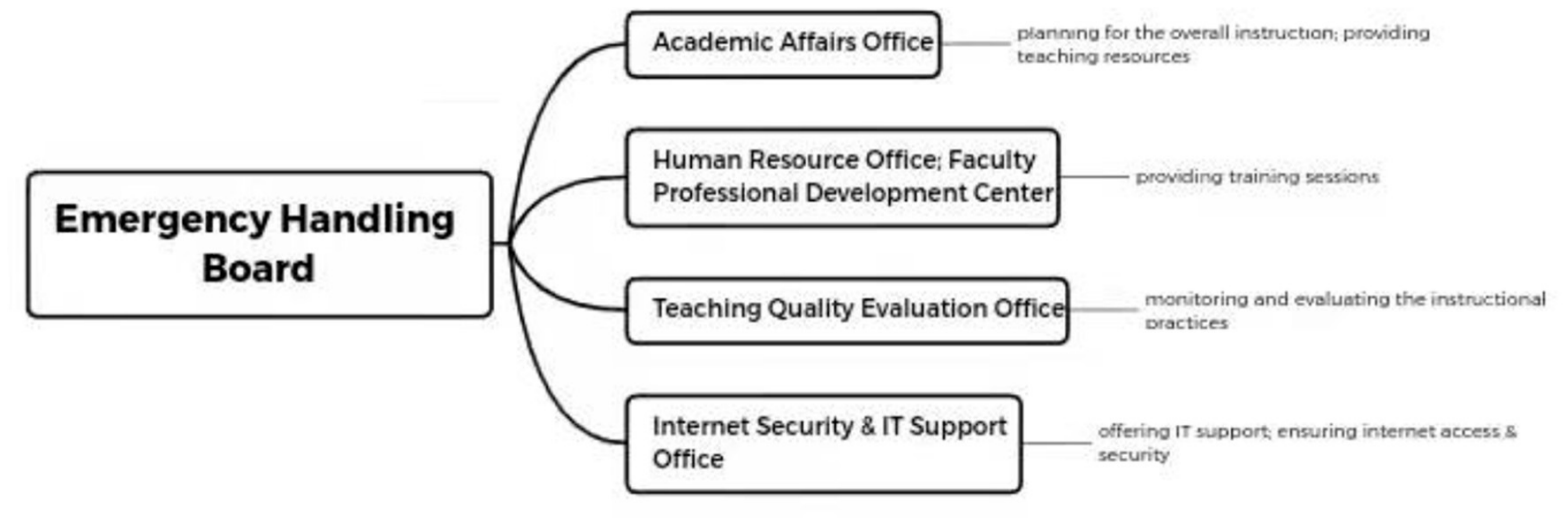
Collaboration from different offices.

However, different subordinate offices responded to and acted differently within universities. Specifically, many Academic Affairs Offices took charge of the overall instruction; Human Resource Offices usually with faculty professional development centers provided online training sessions to teachers and instructors. IT Support and Internet Security Offices gave sufficient support to faculty and teachers and helped them to solve technical issues. Teaching Quality Evaluation Offices monitored and evaluated the instructors' practices (see Figure 2).

In addition, these administrative, subordinate offices to the EHB played uneven roles across different universities. For example, in some universities (e.g., sites 1, 2, and 4), the EHBs collected all ideas and proposed actions from different subordinate, administrative offices and then finalized the polices. However, at other universities (e.g., sites 3, 5, 6, and 7), the EHBs monitored these subordinate, administrative offices and empowered them to issue timely policies facilitating instruction. For example, site 1 was the only site that had created a separate webpage for its EHB, which announced and listed all policy guiding online instruction during the pandemic. At other sites, instructional policies might be viewed *via* different means from other subordinate, administrative offices.

#### The Implementation Stage: How Institutional Schools, Departments, and Teachers Reacted to the Policies

##### Adequate Teaching Resources Recommended

E-learning or online instruction cannot be fully operated or delivered without the incorporation or facilitation of effective instructional platforms or technological tools. As one of the initiatives, the subordinate, administrative offices provided instructors or teachers with substantial teaching resources. In this study, we found that all universities or institutes provided their instructors with three primary sources of online instruction resources, namely, Chinese MOOCs, instructional platforms, and technological tools. For the Chinese MOOCs, two primary platforms, including Chinese MOOC and XuetangX, were offered to instructors. These platforms offered dozens of open-source courses, allowing instructors to choose whatever fit their target courses. The second type of teaching resource was instructional platforms or learning management systems (LMS) specifically created for instructional and evaluative purposes. For example, *Blackboard* was a widely used LMS across different universities. The last type of the teaching resource was social media software, which were used for live teaching and communication purposes. Technological tools, including ZOOM, Tencent Meeting, QQ and WeChat fell into this category and were widely used across the different universities. In this study, most universities provided instructors with access to the Chinese MOOCs and LMSs. Then, instructors could choose whichever platform they found easiest or most effective to operate. However, at some universities (e.g., sites 4 and 5), certain LMSs were mandated for teaching evaluation purposes.

##### Timely Professional Training Offered

**Figure 3:**
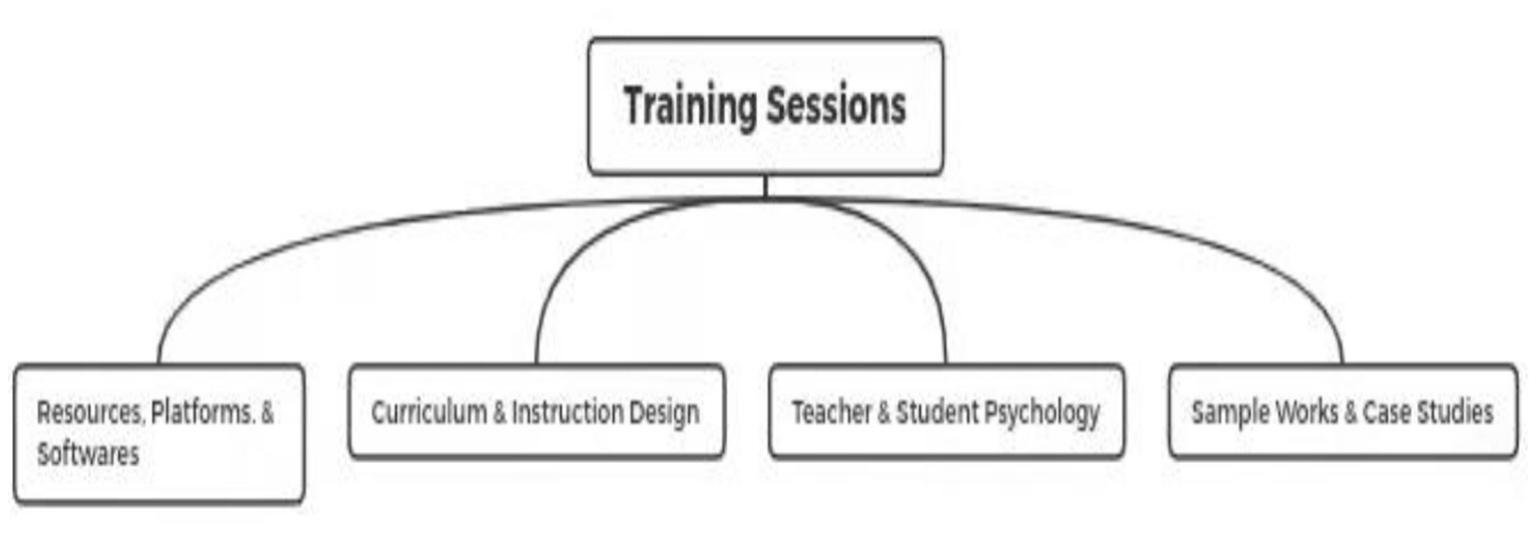
Sufficient training provided.

In addition to teaching resources, universities in this study provided their instructors with sufficient training sessions to smooth over challenges or difficulties in using teaching resources. In a further step, this initiative guaranteed the quality and operation of online instruction during the pandemic. For example, from the beginning of February to the middle of May, site 5 held 14 online training sessions for all faculty members and instructors, all sessions being offered from the Human Resources Office. Likewise, sites 2 and 4 held 10 and 18 training sessions respectively for their instructors. Generally, these sessions fell into one of four major categories, including resources, platforms and software, curriculum and instruction design, teacher and student psychology, and sample works and case studies. These categories complemented one another allowing instructors to smooth over challenges and difficulties in their instructional practices (see Figure 3).

##### Effective Technology Support Provided

Teaching resources and teacher training worked in tandem to guarantee the curriculum design of online instruction; however, chances remain for instruction to be interrupted based on unexpected technical issues or unstable internet access. In the current study, we found all universities were supported by a specific unit or office pertinent to internet security and IT support. These offices offered continued help and support to all teachers across all disciplines online. They guaranteed a smooth online context for teachers to design and implement their curricula and lesson plans. They also supported FTDC in giving lectures or training on how different technologies and techniques may support online instruction during the pandemic. At some universities (e.g., sites 1, 2, and 4), we also found that IT support offices assigned their staff to individual schools or departments and provided full-time support during the pandemic.

#### The Evaluation Stage: How Administrators and Teachers Monitor and Reflect on the Process

##### Teaching Performance Monitored

With all teaching resources, training sessions, and IT support offered, curriculum design and implementation during the pandemic was technically sound and safe. However, to ensure teaching quality, certain departments or offices at these selected universities were appointed to monitor teacher performance. In this study, we found that the monitoring process occurred typically at the beginning of the Spring semester; however, not all universities employed the monitoring process. At sites 1, 3, 5, and 6, the EHBs did assign certain offices or staff to monitor the process; at other sites, however, the EHBs chose to leave the instructors some wiggle room, asking them to concentrate on their curriculum design and instruction first, rather than sacrifice their time on evaluation.

##### Teaching Quality Assured

With all the policies, training, and support from different offices and centers, another team that worked to ensure the quality of teaching during the pandemic was the Development Planning and Quality Management Office, in which TQAT was located. TQAT monitored and evaluated the instructional practices during the pandemic, typically during the mid- and final-term. TQAT assigned the monitoring and evaluative tasks to different teaching supervisors and set up evaluative rubrics for instructors' performance. With the rubric, supervisors evaluated instructors' performance and gave timely feedback and responses to instructors. In this study, we found some universities (e.g., sites 1 and 5) gradually placed increased evaluation scrutiny on their instructors, typically scheduling relatively complicated evaluation procedures at the end of the Spring semester.

### Tasting the PIE: Teacher Perception of the Educational Planning Policies

In response to RQ2, we interviewed eight participants from the selected sites and universities and collected their perceptions of the educational planning policies their universities or institutes had issued during the first round of COVID-19. As we are metaphorically comparing the educational planning process to the baking of the PIE, we regard the teacher perceiving process of the educational planning policies as how the teachers might taste and comment on the PIE. Generally, we found that the teachers and instructors across all sites affirmed the educational planning policies but also pointed out how the educational planning policies might be improved in the future, typically in the post-pandemic future where a pandemic may occur at any time or even carry on indefinitely. We also reported teacher preference of traditional, face-to-face classes over online classes and summarized their reflections in language education during the pandemic.

#### Overall Confirmation and Appreciation of the Educational Planning Policies

The participants in the study all affirmed the contribution of their universities in maintaining a smooth curriculum during the pandemic. This is consistent with the existing literature where large samples of teachers across the nation were surveyed for their appraisals (e.g., Zheng et al., 2020). All participants reported they had received sufficient training, adequate teaching resources, and IT support from their affiliates and had managed to deliver instruction. Participant 4 in the study reported her perceptional shift and stated that she became proud of, and even confident in, her affiliate when dealing with unexpected instruction during the pandemic. Likewise, Participants 1, 2, and 5 all reported that their respective affiliates had responded efficiently to the emergency.

#### Improvements for Post-pandemic E-Learning Instruction

Alongside overall affirmation, some participants also reported certain improvements that their respective affiliates or universities may consider for future educational planning. For example, participants 4 and 5 believed their affiliate may consider sticking to their originally proposed evaluation criteria instead of changing these criteria repeatedly. They reported how their affiliate increased the instructors' load in preparing for the second-half semester evaluations. Also, some participants, e.g., participants 1, 2, 5, and 8 argued that the training they had received might be overcomplicated or superfluous. They argued that the training department should survey their instructors' needs in future to save time and energy in planning these training sessions. They also reported that survey processes would help the training department schedule other necessary training sessions focused on teacher emotions during the pandemic.

#### Preference to Traditional Instruction and Face-to-Face Communication

While the participants affirmed the educational planning policies and even offered suggestions for future planning improvements, most of the participants still preferred traditional, face-to-face class over online classes. Specifically, participants 1, 2, 3, 4, 5, and 8 reported that online instruction had increased their workload in preparing for and evaluating lectures, their anxiety in delivering online classes, and their grading work. For example, participants spent extra time completing training sessions to learn about different platforms and technological systems. Due to unstable network or Internet access issues, direct interaction and communication with students was sometimes paused during lectures, which caused both instructor and student depression and anxiety.

#### Reflection on Language Education During the Pandemic

The participants in the study also reflected on their teaching during the pandemic and reported accordingly. As most participants are language teachers and linguistic professors, they reported that online instruction plans fit the subject matter of their courses. Typically, advanced technological tools helped them with the systemic evaluation of their students' performance, typically for language skill-based performance. For example, participant 3 reported that the Speedex software he had used was quite effective and efficient in setting up reading and listening assignments and measuring student performance. Participant 6 also reported that the technology or platform used in her writing class had saved her maximal time in giving student feedback. However, these participants also reported their doubts or concerns over the aptness of educational planning policies in other types of courses, including graduation project design, experiments, and practicums.

## Discussion

We have presented how national and institutional policies have facilitated online instruction during the pandemic at sampled universities. Gao et al. (2021) summarize four points that would maximize the operation and implementation of online courses during an un-precedented time, typically in the higher education. These points include the efficiency of delivering policies, the cooperation among different offices and schools across campuses, the sufficiency of technological and pedagogical training to appease faculty members and teachers both physically and mentally, and the appropriateness of monitoring. Findings of this study regarding educational planning policies were consistent with existing literature (e.g., Yadav et al., 2021).

### Teacher Agency in the Emergency Remote Teaching and Planning

While educational planning policies provided teachers with a great recipe to bake the PIE, we found that teacher collaboration and teacher agency were important in making the baking process possible. Teacher collaboration has long been a crucial concept for faculty capacity building (Datnow and Park, 2018) and student engagement and achievement (Hitt and Tucker, 2016). Collaboration among educators and education systems became especially salient during the pandemic (Merga et al., 2021). If collaboration provided online instruction with an external smoothening environment, then teacher agency drove online instruction in the direction that educators and teachers had planned. Chen (2022) reported how online instruction in unprecedented times had managed to help teachers exert more agency than in traditional classrooms. Given the findings of this study, we believe that extra learning hours for different platforms and software, enlarged workloads to design and prepare lectures, and increased concerns and anxiety in engaging students online indeed call for greater teacher agency and effort during a pandemic.

### Tensions Between Teacher Beliefs and Practices

However, we also found tensions between teacher perception of educational planning policies and the direction for which these policies had been planned. This is consistent with existing literature (e.g., Merga et al., 2021; Salas-Rueda et al., 2022). Teacher perception and practices are in nature in complex, fostered by different ideological, political, economic, and sociocultural affordances (Gao, 2021b) even in normal times. We believe these tensions to be valuable in guiding the direction of future educational planning during a pandemic or disaster times.

The findings of these tensions also offered insights to teacher educators on how to improve professional development programs through reviewing teachers' beliefs and practices in a complex, but pragmatic manner. Gao (2021a) argued that teachers' beliefs and practices used to be examined in a linear way with either consistency or inconsistency between these two constructs in 1990's. Gradually, these constructs were examined through a pragmatist paradigm when they were proved to appear in complex, non-linear, and unpredictable relations. With an increasing number of constructs (e.g., applications and software) or learnings (e.g., pedagogies and theories) swarming into teachers' life, their beliefs and practices are connected consistently, at least in the very first stages when teachers meet with these constructs or learnings. Therefore, we may re-consider the pursuit of the consistency between teachers' beliefs and their practices typically in the Chinese culture (*Zhi Xing He Yi*), given the fact that inconsistency may push teachers out of their comfort zone and further develop themselves.

### Transfer From Emergency Remote Teaching to Regular, Sustainable Online Teaching

As a new way of imparting knowledge, online instruction boasts unique advantages and will thrive with the following added measures: For the institutions, suitable instructional platforms, timely professional training, and effective technology support should be provided for the teachers. For supervisors, the monitoring process and evaluation approaches should be carried out in a way that will not dampen teacher enthusiasm nor affect their performance. For teachers, priority should be given to online, rather than traditional classroom learning.

The findings of the study yielded insights to teacher educators and also inservice teachers that we have to steer our beliefs toward accepting e-learning would be something regular and normalized in the future, due to the long, unpredictable pandemic or disaster. Therefore, shift from emergency remote teaching to a regular, sustainable online teaching mode would become something inevitable. As repeatedly stated in the study, the difference between ERT and e-learning lies in the quantity and quality of the educational planning. We thus believe that teachers and teacher educators gain insights from the ERT experiences during the current pandemic and make them inform them of their future planning and teaching. More efforts in conceptualizing and theorizing ERT are still required for future schooling and education.

## Conclusion

We would like to highlight what we can learn from COVID-19 to conclude this paper. The unprecedented COVID-19 pandemic has caused our schooling to move in a new direction. Where we used to think e-learning would be something supplementary to the traditional classroom, we now believe it might be a replacement or at least a competitive counterpart to traditional instruction. Therefore, educational planning policies for online instruction, particularly during disasters or pandemics, is something of great value and importance to educators and scholars currently working in academia. However, educational planning and teacher perception and practices are complex, with interwoven connections and tensions. Therefore, we explored the educational planning and policies in China during the first round of COVID-19 and report how these policies helped maintain smooth and successful online instruction during the pandemic. In addition, we looked at teacher perception of these planned policies at sampled universities and how teachers appreciated and appraised these policies, which offered insights to studying academic resilience, teacher perception, and educational planning during disasters or pandemics.

Our study has its own limitations. Firstly, it is not generalizable to all contexts, as it is restricted to science and technology-based universities in China. Besides, all participants are under forty. For future research, we expect cross-national, comparative studies in similar mixed methods approaches to be conducted. Findings and insights gained from such studies would contribute to improving global academic teaching practices by helping our educators plan online instruction even in post-pandemic times.

## Data Availability Statement

The raw data supporting the conclusions of this article will be made available by the authors, without undue reservation.

## Ethics Statement

Ethical review and approval was not required for the study on human participants in accordance with the local legislation and institutional requirements. The patients/participants provided their written informed consent to participate in this study.

## Author Contributions

YG has contributed to the overall design, organization, and logic of the paper. GZ and YW have contributed to the organization, design, and implementation of the study. YG, YW, AK, and XW have contributed to the data collection and analysis of the paper. All authors have contributed to the drafting, revising, and proofreading of the paper. All authors contributed to the article and approved the submitted version.

## Funding

This study was primarily funded through Dalian Maritime University (Grant No. 02500805), 2019–2022, Central Universities China (Grant No. 3132022332), 2022–2023, the 13th-Five-Year Education Planning Project of Liaoning Province (Grant No. JG20DB053), 2020–2021, Liaoning Provincial Federation Social Science Circles (Grant No. 2022slqnwzzkt-016), 2022, and Foreign Language Teaching and Research Committee, China Association of Higher Education (Grant No. 21WYJYZD04), 2021. This study was also sponsored by the Teacher Education Project of Henan Provincial Education Department, entitled “Using Online Learning Resources to Promote/Enhance English as a Foreign Language Teachers' Professional Development in the Chinese Middle School Context” (Grant No. 2022-JSJYYB-027). This study was also funded through the New Liberal Arts Research and Reform Practice Project of the Ministry of Education (Grant No. 2021100027) and the Liaoning Provincial Foundation of Social Sciences (Grant No. L17BYY015).

## Conflict of Interest

The authors declare that the research was conducted in the absence of any commercial or financial relationships that could be construed as a potential conflict of interest.

## Publisher's Note

All claims expressed in this article are solely those of the authors and do not necessarily represent those of their affiliated organizations, or those of the publisher, the editors and the reviewers. Any product that may be evaluated in this article, or claim that may be made by its manufacturer, is not guaranteed or endorsed by the publisher.
